# In Vivo Imaging of Neuronal Activity Shifts in the Somatosensory Cortex After Morphine Tolerance in Mice

**DOI:** 10.1111/adb.70155

**Published:** 2026-04-14

**Authors:** Guangyan Zhang, Weiwei Meng, Ling‐Jun Xu, Bin Mou, Zongqin Xiang, Xiangcai Ruan, Yong U. Liu, Haihua Shu

**Affiliations:** ^1^ Department of Anesthesiology, Guangdong Provincial People's Hospital (Guangdong Academy of Medical Sciences) Southern Medical University Guangzhou China; ^2^ Department of Anesthesia and Pain Medicine, The Sixth Affiliated Hospital Sun Yat‐Sen University Guangzhou China; ^3^ Biomedical Innovation Center, The Sixth Affiliated Hospital Sun Yat‐Sen University Guangzhou China; ^4^ Department of Pain Management The Affiliated Guangdong Second Provincial General Hospital of Jinan University Guangzhou Guangdong China; ^5^ Laboratory for Neuroimmunology in Health and Disease, Center for Medical Research on Innovation and Translation, The Second Affiliated Hospital, School of Medicine South China University of Technology Guangzhou China; ^6^ Department of Neurology, Multi‐Omics Research Center for Brain Disorders, The First Affiliated Hospital University of South China Hengyang China

**Keywords:** morphine, neuronal activity, primary somatosensory cortex, tolerance, two‐photon

## Abstract

Morphine is widely used to treat severe pain, but its analgesic effect diminishes with repeated use due to the development of tolerance. Reversing this tolerance remains a clinical challenge, as its underlying mechanisms are complex and not fully understood. Although the involvement of multiple central nervous system regions in morphine tolerance has been established, the role of the primary somatosensory cortex (S1)—a key region for sensory perception—remains unclear. In this study, we used in vivo two‐photon calcium imaging to longitudinally track neuronal activity in S1 during the induction of morphine tolerance in mice. Mice received daily morphine injections (10 mg/kg, s.c.) for 7 days. Behavioural assays confirmed the development of tolerance, as shown by diminished analgesic responses. While total neuronal activity in S1 remained stable after the first morphine injection, a significant increase was observed on Day 7. At the single‐neuron level, three response patterns were identified: increased, decreased and stable firing following morphine administration. Notably, these subpopulations were dynamically restructured after tolerance was established. Our findings reveal that morphine tolerance is accompanied by network‐level reorganization in the somatosensory cortex, suggesting a cortical contribution to altered sensory processing during chronic opioid exposure.

## Graphical abstract

Morphine tolerance drives reorganization of neuronal activity in the somatosensory cortex.

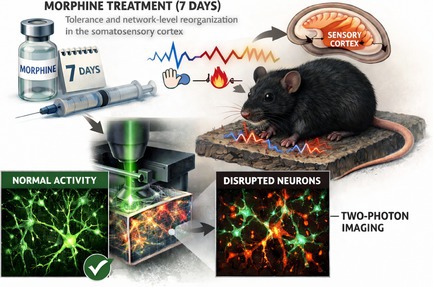

## 1 Introduction

Morphine is a potent opioid analgesic commonly used to manage severe pain. However, with prolonged administration, its effectiveness diminishes due to the development of analgesic tolerance. Clinically, this often necessitates escalating doses to achieve the same level of pain relief, increasing the risk of severe side effects such as respiratory depression and dependency [1]. Understanding the mechanisms underlying morphine tolerance is critical for developing safer and more effective pain therapies.

Previous studies have identified a range of mechanisms contributing to opioid tolerance, including receptor‐level changes such as *μ*‐opioid receptor (MOR) desensitization and internalization, as well as neuroimmune interactions involving microglia and proinflammatory cytokines [2]. While much of this research has focused on the spinal cord and brainstem, recent attention has turned to cortical regions, including the primary somatosensory cortex (S1). This area plays a critical role in processing nociceptive inputs and mediating pain perception, and emerging evidence suggests that S1 is sensitive to opioid modulation [3].

Functional MRI studies have shown overlapping activation patterns in S1 during both pain perception and opioid analgesia [4]. Electrophysiological recordings further demonstrate that morphine can enhance both spontaneous and stimulus‐evoked neuronal activity in S1 [5]. However, little is known about how these activity patterns evolve during chronic morphine exposure and whether S1 undergoes network‐level changes that contribute to the development of tolerance.

Two‐photon calcium imaging offers a powerful method for monitoring neuronal activity at single‐cell resolution across days in awake, behaving animals. In this study, we used a head‐fixed, freely moving imaging system to perform longitudinal recordings of calcium dynamics in Layer II/III neurons of S1 in mice undergoing repeated morphine administration [6]. By combining behavioural assays, immunohistochemistry and cellular imaging, we aimed to investigate how morphine tolerance affects both global and single‐neuron activity patterns in the somatosensory cortex.

## 2 Materials and Methods

### 2.1 Animals

Eight‐week‐old male C57BL/6J mice were obtained from the Guangdong Medical Laboratory Animal Center. All animal procedures were approved by the Ethics Committee of Guangdong Provincial Hospital (KY2024‐894‐01) and conducted in accordance with institutional guidelines.

### 2.2 Morphine Tolerance Model and Behavioural Assays

Morphine tolerance was induced via daily subcutaneous injections of morphine sulphate (10 mg/kg, 2 mg/mL) for seven consecutive days. To assess analgesic responses, mechanical sensitivity was measured using von Frey filaments applied to the plantar surface of the hind paws, both before and 40 min after morphine administration. The ‘up‐down’ method was used to determine mechanical thresholds. Control mice received equivalent volumes of saline. Thermal nociceptive thresholds were assessed using the hot‐water tail‐immersion assay. Mice were gently restrained, and the distal 2–3 cm of the tail was immersed in a water bath maintained at 50.0°C ± 0.5°C. A cut‐off time of 15 s was applied to prevent tissue damage. Analgesic effects were expressed as the percentage of maximum possible effect (MPE%), calculated as

$$\begin{array}{c} \mathrm{MPE}\%=\left[\left(\text{test latency}—\text{baseline latency}\right)/\right.\\ \left.\left(\mathrm{cut}-\mathrm{off}\;\text{time}—\text{baseline latency}\right)\right]\times 100\%. \end{array}$$

Baseline latencies were measured twice prior to treatment and averaged for each animal.

### 2.3 Surgical Procedure and Virus Injection

Surgery was performed under 1%–1.5% isoflurane anaesthesia with the animal positioned in a stereotaxic frame. After antiseptic preparation, a circular craniotomy (3 mm in diameter) was performed over the right primary somatosensory cortex (S1; coordinates: AP − 1.5 mm, ML + 1.9 mm relative to bregma). A glass micropipette was used to inject 300 nL of AAV‐hSyn‐jGCaMP7f (BrainVTA, Lot No. PT‐1887‐231 017) into cortical Layer II/III at a depth of 250 μm. The injection was delivered at a rate of 1 nL/s, and the pipette was left in place for an additional 5 min after injection to allow for viral diffusion.

Following haemostasis, the cranial window was sealed with a 4‐mm glass coverslip using light‐curable dental cement (Tetric EvoFlow). A metal head ring (SITRANTECH, China) was affixed to the skull for future head fixation to a mouse treadmill (SITRANTECH, China) during imaging. Post‐operative recovery was monitored on a heated pad. Mice were returned to their home cages for 3 weeks to allow sufficient expression of the calcium indicator. Expression levels were verified using two‐photon microscopy. Only mice with robust cortical expression were selected for imaging experiments.

### 2.4 Two‐Photon Calcium Imaging in Awake Mice

Imaging was conducted using a Leica two‐photon microscope with excitation set at 920 nm. Laser power was maintained at 3 W. Recordings were acquired from cortical Layer II/III at depths of 150–200 μm in awake, head‐fixed mice. A custom imaging platform allowed for head fixation while permitting voluntary locomotion on a treadmill. Baseline neuronal activity was recorded for 15 min. Immediately following subcutaneous morphine injection (10 mg/kg), the same field of view was imaged continuously for an additional 45 min. Recordings were conducted on days 1, 3, 5 and 7 to monitor longitudinal changes in neural activity.

### 2.5 Image Processing and Calcium Signal Analysis

Motion correction and region of interest (ROI) detection were performed using Suite2P. Fluorescence data were analysed in MATLAB and Python. ΔF/F_₀_ was calculated as: ΔF/F_₀_ = (F_c_ − F_₀_) /F_₀_, where F_c_ = F − 0.7 × F_neu_ + 0.7 × F_neu_median_, F is the raw fluorescence signal, F_neu_ is the surrounding neuropil signal, and F_neu_median_ is the median neuropil fluorescence. F_₀_ was defined as the median F_c_ value.

### 2.6 Calcium Transient Detection

Calcium transients were identified based on the criterion: ΔF/F_₀_ ≥ mean (ΔF/F_₀_) + 1 SD, after Gaussian filtering (*σ* = 1.5) and baseline adjustment using the lowest 10% of signal values. Peaks were detected using the find_peaks function from SciPy. For each transient, boundaries were defined by the nearest local minima. Transient area was computed via Simpson's rule integration, and amplitude was defined as the peak ΔF/F₀ value. For each neuron, the number of transients, transient amplitude, transient area and peak time points were extracted.

### 2.7 Neuronal Response Classification

Based on changes in transient frequency before and after morphine injection, neurons were categorized into three groups: low‐to‐high (≥ 20% increase), high‐to‐low (≥ 20% decrease) and stable (< 20% change).

### 2.8 Immunohistochemistry

Mice were deeply anaesthetised and transcardially perfused with PBS, followed by 4% paraformaldehyde (PFA). Brains were postfixed in 4% PFA overnight, then cryoprotected in 20% and 30% sucrose solutions. Thirty‐micron brain sections were mounted on adhesion slides and washed in PBS. Sections were blocked for 1 h in 5% goat serum and 1% Triton X‐100 in TBS, then incubated with anti‐c‐Fos primary antibody (Cell Signalling Technology, 1:1000) at 4°C for 48 h. After washing, slides were incubated with fluorescent secondary antibodies, counterstained with DAPI and coverslipped. Imaging was performed using the PhenoImager HT 2.0 system.

### 2.9 Statistical Analysis

Data are presented as mean ± SEM Statistical analyses were performed in GraphPad Prism 10. A significance threshold of *p* < 0.05 was used throughout. Specific tests (e.g., paired/unpaired *t*‐tests, ANOVA) are indicated in the corresponding figure legends.

## 3 Results

### 3.1 Morphine Tolerance Enhances Neuronal Activation in the Somatosensory Cortex

**FIGURE 1:**
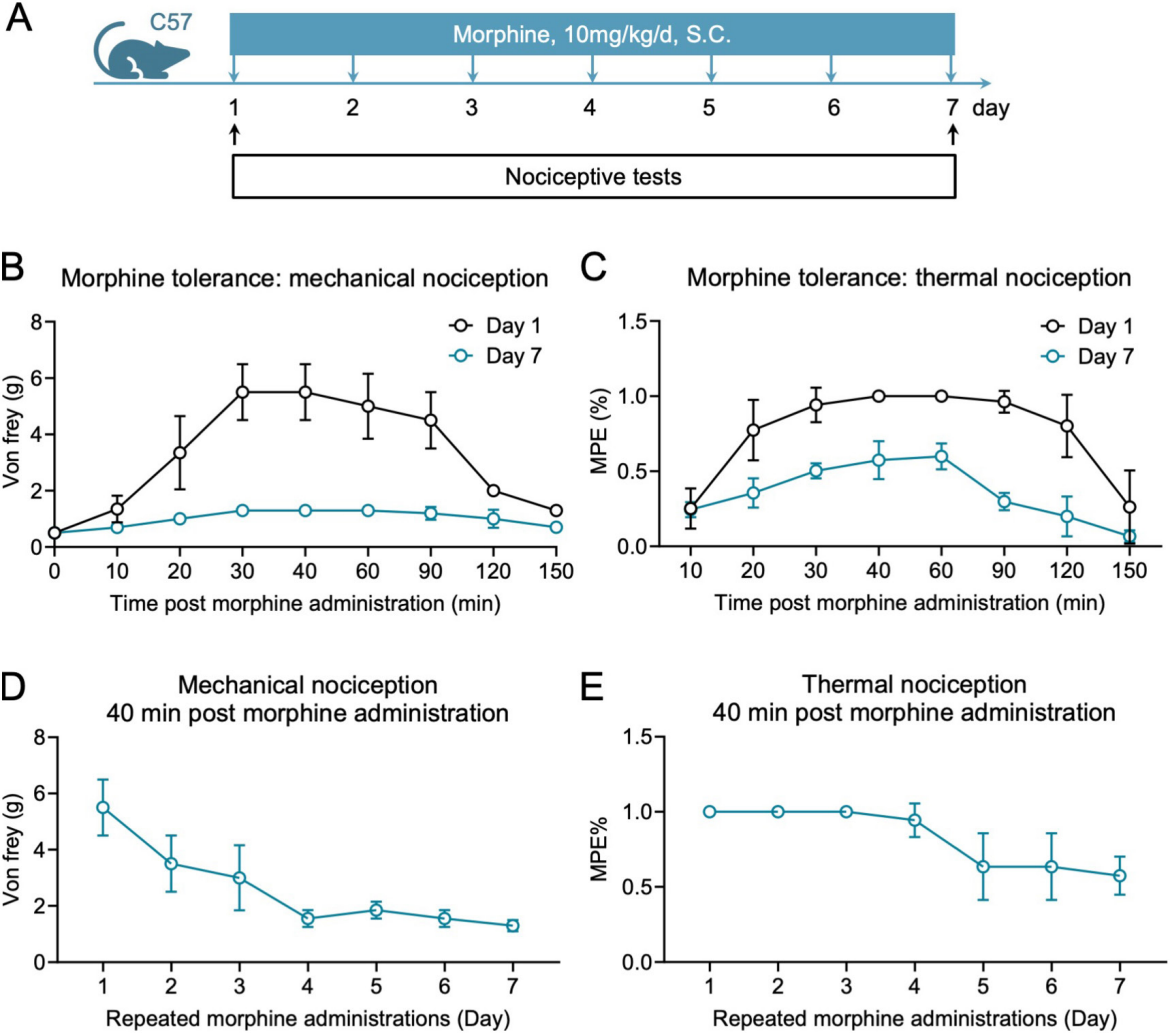
Behavioural assessment of morphine tolerance development in mice(***n*** = 4 per group). (A) Schematic timeline of the experimental protocol for chronic morphine administration (10 mg/kg, s.c., daily for 7 days). (B) Antinociceptive effects and tolerance development assessed by mechanical sensitivity using von Frey filaments. (C) Antinociceptive effects and tolerance development assessed by thermal sensitivity using the tail‐flick assay. (D) Time course of mechanical pain thresholds measured 40 min after morphine injection from Day 1 to Day 7. (E) Time course of thermal pain thresholds measured 40 min after morphine injection across the 7‐day period. Data are presented as mean ± SEM Statistical comparisons were performed using two‐way ANOVA followed by Bonferroni's post hoc test. *p < 0.05, ****p < 0.0005, comparing Day 1 to Day 7.

To confirm the successful induction of morphine tolerance, mice received daily subcutaneous injections of morphine (10 mg/kg) for seven consecutive days. Before and 40 min after morphine administration, mechanical pain response was tested by applying von Frey filaments to the hind paw and thermal pain response was tested by hot water tail flick. Over the 7‐day period, the analgesic effect of morphine progressively diminished (Figure 1A–E). These findings confirm the development of morphine tolerance in this model.

**FIGURE 2:**
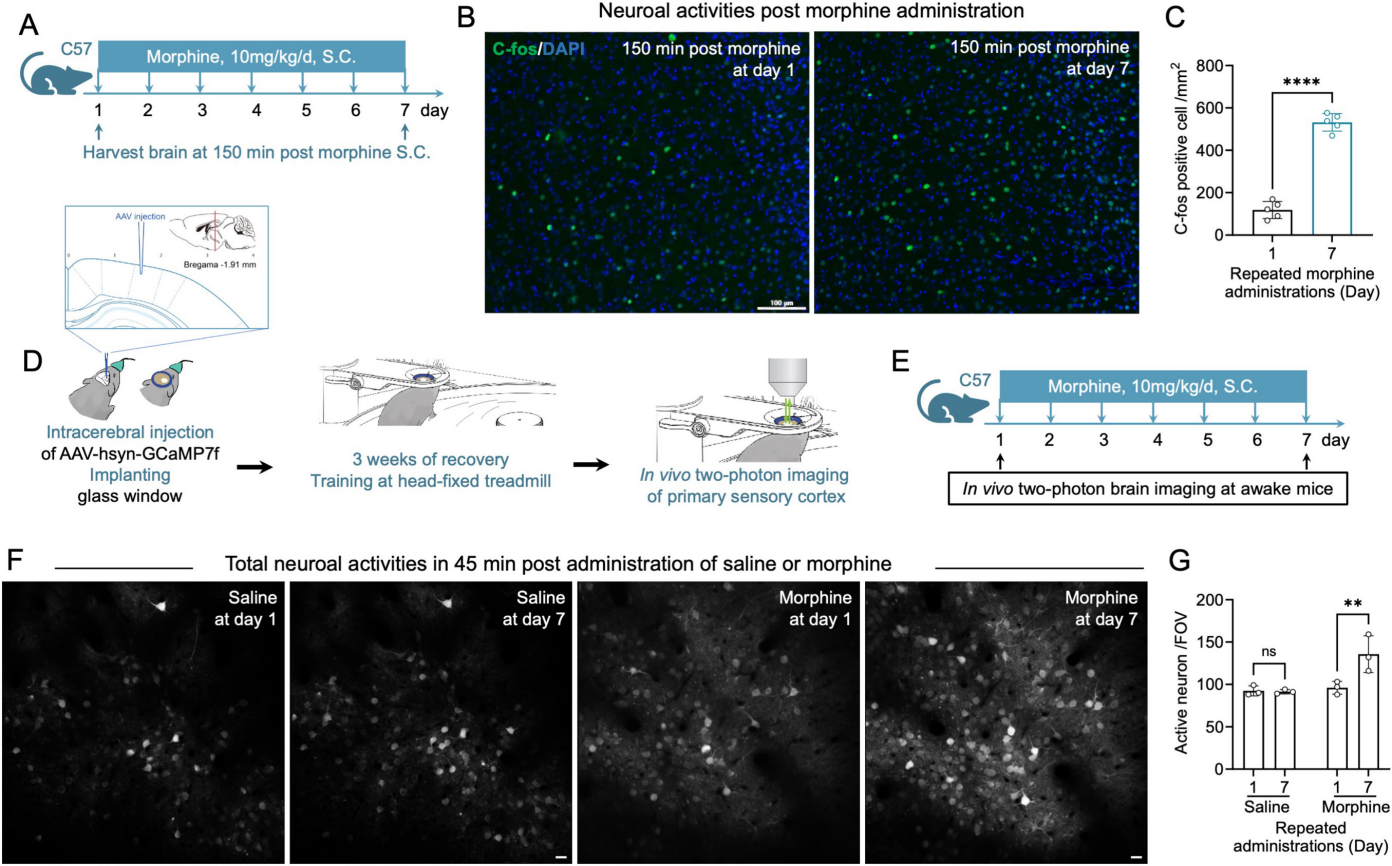
Neuronal excitability in the primary somatosensory cortex increases after morphine tolerance. (A) Experimental timeline illustrating chronic morphine administration and time points for brain tissue collection. (B) Representative c‐Fos immunofluorescence images showing c‐Fos‐positive cells in the primary somatosensory cortex (S1) on Day 1 and Day 7 of morphine treatment. (C) Quantification of c‐Fos‐positive neurons per unit area in S1 across experimental phases (*n* = 5 mice). ****p < 0.0005, comparing Day 1 and Day 7. (D) Schematic showing AAV‐mediated calcium indicator expression and cranial window implantation over S1 (AP −1.9 mm, ML +1.5‐mm relative to bregma). (E) Timeline for in vivo two‐photon calcium imaging in awake mice during morphine administration. (F) Representative two‐photon images of neuronal Ca^2+^ activity in S1 on Day 1 and Day 7 in saline‐ and morphine‐treated mice. (G) Quantification of active Ca^2+^‐expressing neurons in S1 on Day 1 and Day 7.

To examine the involvement of the primary somatosensory cortex (S1) in morphine tolerance, we assessed neuronal activation using c‐Fos immunohistochemistry. Expression of c‐Fos—a marker of recent neuronal activity—was significantly elevated in S1 of morphine‐tolerant mice compared to saline‐treated controls (Figure 2A–C), suggesting that repeated morphine exposure enhances cortical excitability.

We next investigated dynamic changes in neuronal network activity using longitudinal in vivo two‐photon calcium imaging in awake, head‐fixed mice (Figure 2D,E). In saline‐treated controls, the number of active neurons in S1 remained stable between Day 1 and Day 7. However, in morphine‐treated mice, a significant increase in active neuron count was observed by Day 7 (Figure 2F,G), further indicating that chronic morphine exposure enhances S1 excitability over time.

### 3.2 Chronic Morphine Administration Induces Shifts in Global and Single‐Neuron Activity

**FIGURE 3:**
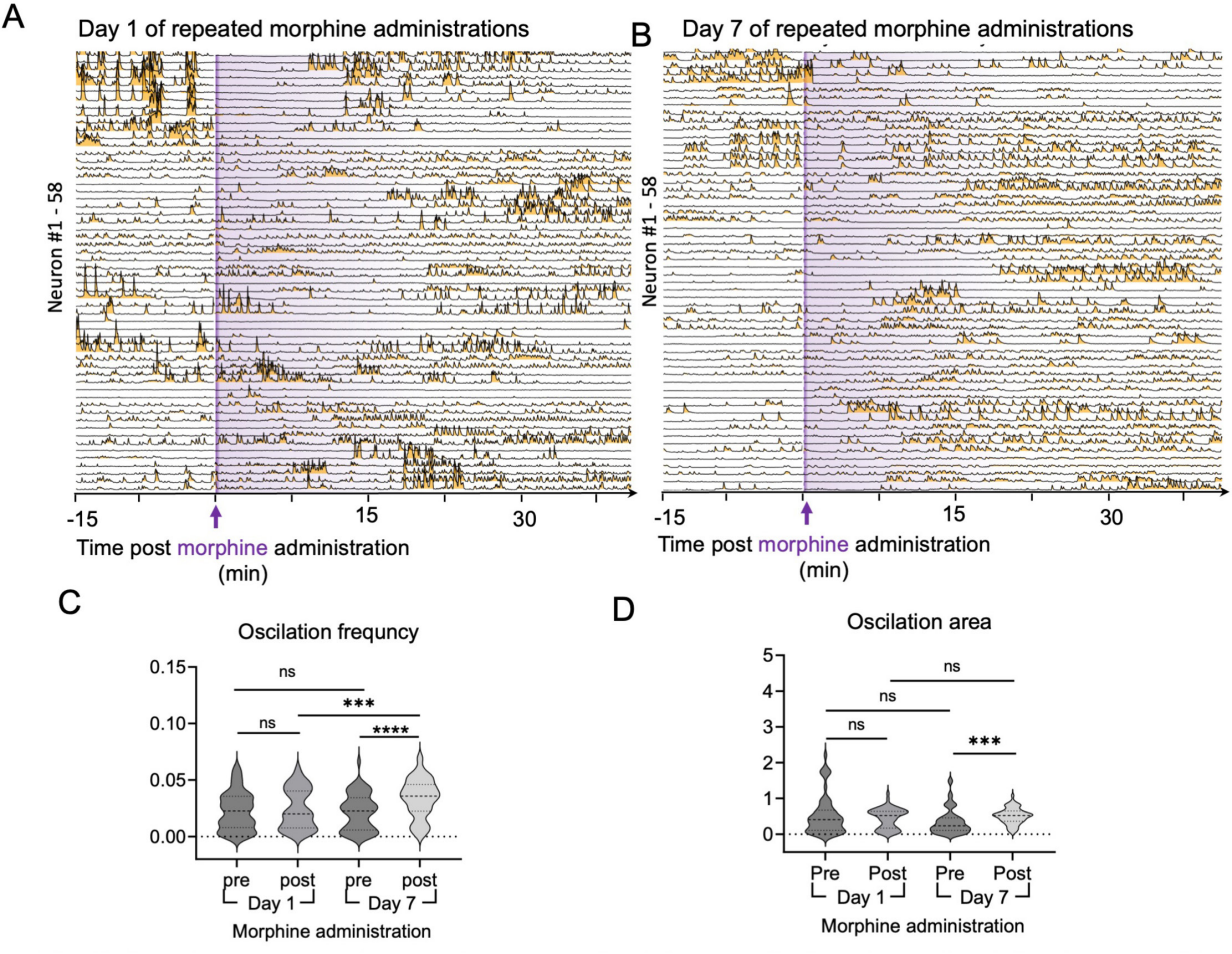
Time‐dependent changes in neuronal calcium dynamics following morphine administration. (A) Neuronal calcium activity (ΔF/F) traces from the same field of view before and after morphine injection on Day 1. (B**)** Neuronal calcium activity (ΔF/F) traces before and after morphine injection on Day 7. (C) Quantification of calcium oscillation frequency from matched neurons before and after morphine injection on Day 1 and Day 7 (*n* = 5 mice). ***p < 0.005, ****p < 0.001. (D) Area under the curve (AUC) per second of calcium oscillations from paired neurons on Day 1 and Day 7 (*n* = 5 mice). ***p < 0.005.

To investigate morphine‐induced changes in neuronal network dynamics at single‐cell resolution, we analysed calcium activity in individual S1 neurons (Figure 3A,B). On Day 1, acute morphine administration produced no significant changes in firing frequency or calcium signal amplitude, suggesting a limited immediate effect on cortical activity. In contrast, by Day 7, both the average firing frequency and area under the calcium transient curve significantly increased after morphine injection (Figure 3C,D), indicating that repeated morphine exposure induces hyperexcitability and functional reorganization in the S1 network.

**FIGURE 4:**
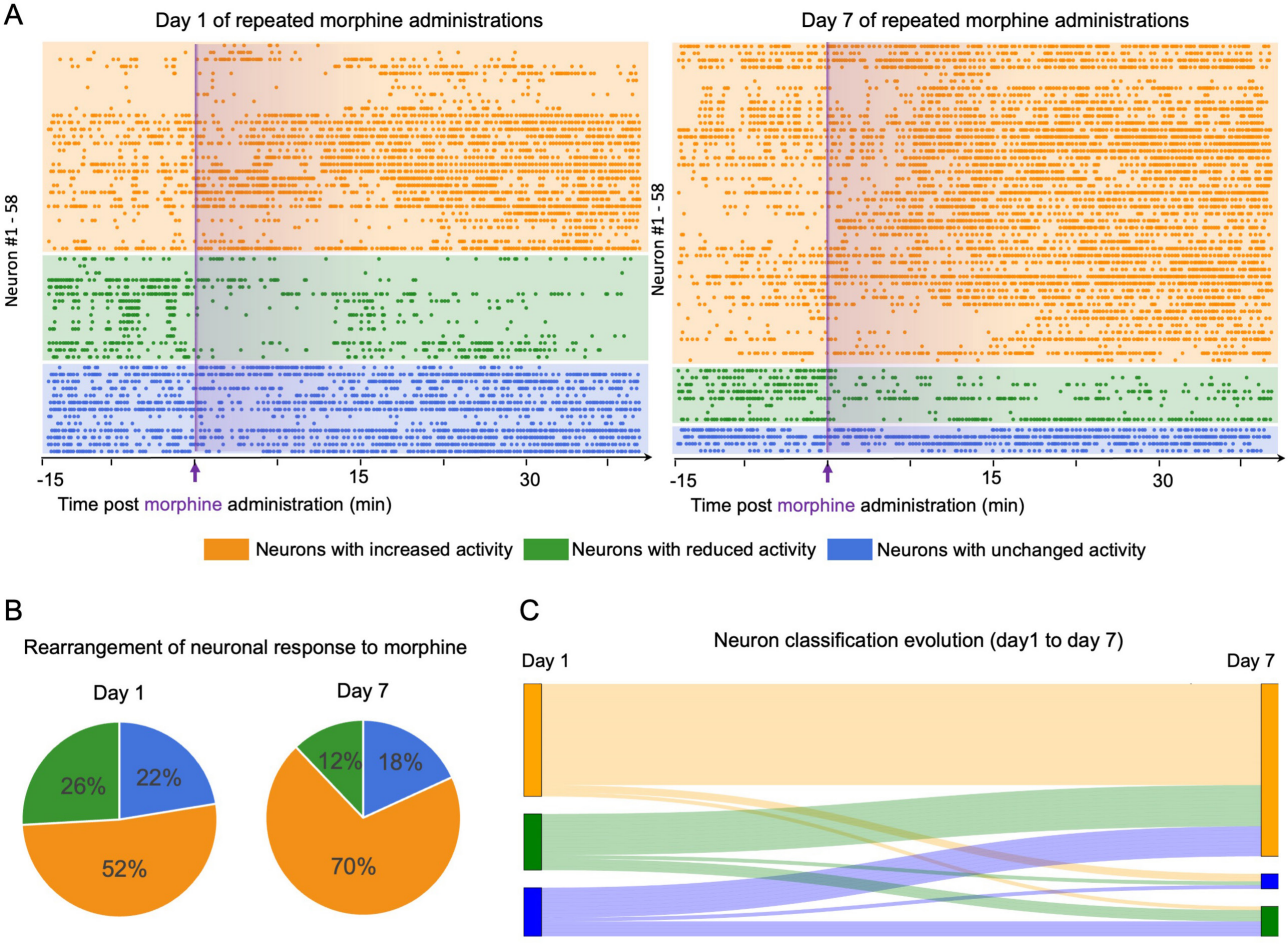
Individual neurons exhibit dynamic category transitions following morphine tolerance development. (A) Classification of neurons based on changes in firing frequency before and after morphine administration on Day 1 and Day 7. Neurons were categorized as: stable (blue), high‐to‐low frequency (green), and low‐to‐high frequency (yellow). (B) Pie charts illustrating the proportional distribution of neuronal response categories on Day 1 and Day 7, highlighting the dynamic reorganization of response patterns. (C) Sankey diagram depicting transitions of individual neurons between categories from Day 1 to Day 7, demonstrating functional reclassification during morphine tolerance.

To explore the heterogeneity of neuronal responses to morphine, we classified neurons into three categories based on changes in calcium transient frequency: low‐to‐high (increased), high‐to‐low (decreased) and no significant change (stable). On Day 1, 52% of neurons exhibited increased activity, 26% showed decreased activity, and 22% remained stable (Figure 4A,B), revealing the diversity of morphine's effects across individual neurons in S1.

We then examined how these categories evolved over time by tracking the same neurons from Day 1 to Day 7. The proportion of low‐to‐high neurons increased from 52% to 70%, while the high‐to‐low group decreased from 26% to 12%. The stable group showed a modest reduction from 22% to 18% (Figure 4B). Notably, a subset of neurons transitioned from the high‐to‐low to the low‐to‐high category, indicating dynamic functional reclassification. This redistribution may reflect network‐level plasticity or an imbalance in excitatory and inhibitory signalling associated with morphine tolerance.

### 3.3 Morphine Tolerance Further Reduces Neuronal Synchronization

**FIGURE 5:**
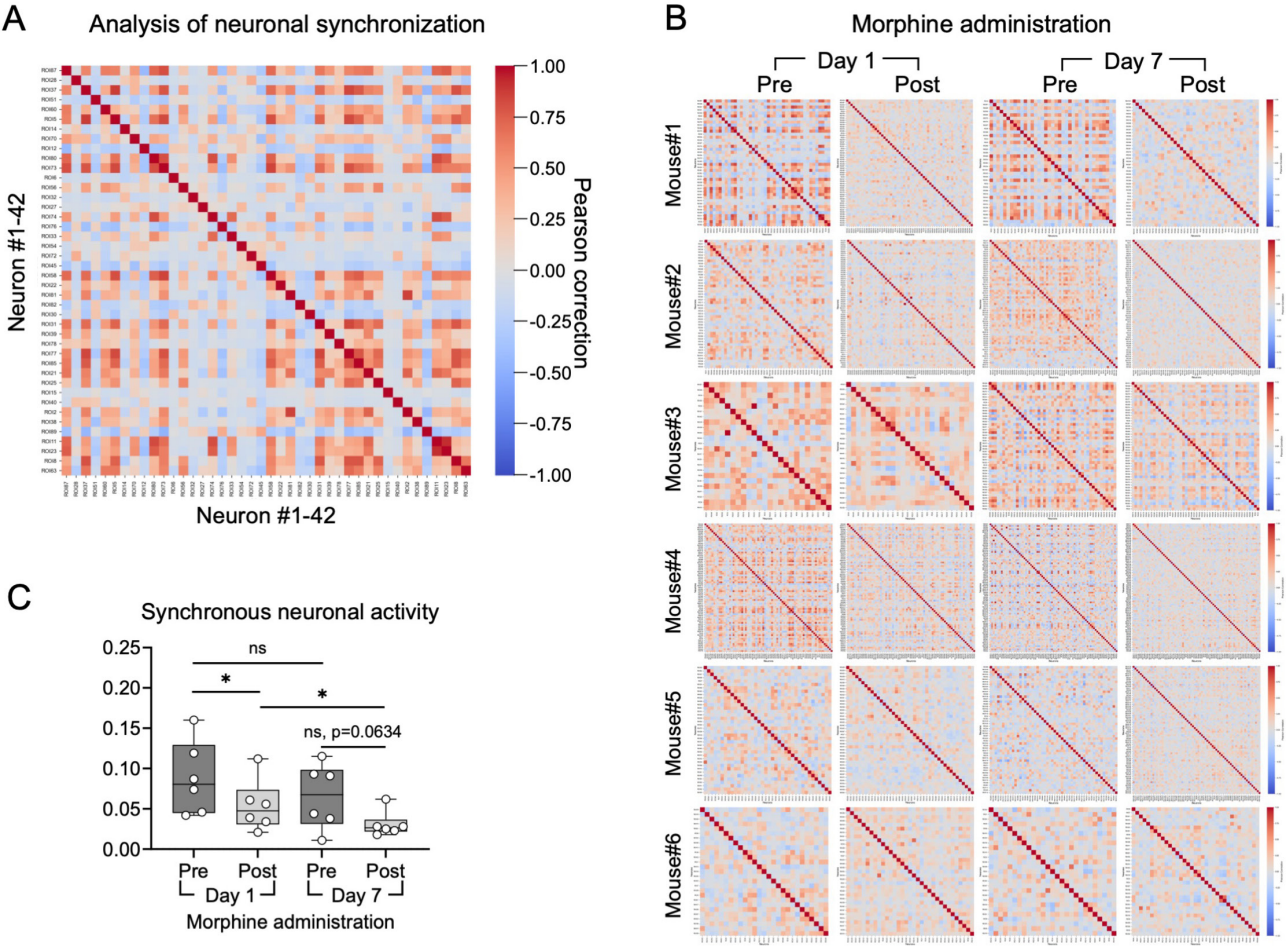
Morphine tolerance exacerbates the reduction in neuronal synchronization following morphine administration. (A) Representative heat map showing pairwise neuronal synchronization in the primary somatosensory cortex, based on temporal Pearson correlation coefficients between individual neuron activity traces. (B) Heat maps of neuronal synchronization from six mice, recorded before and after morphine administration on Day 1 and Day 7. (C) Quantification of mean neuronal synchrony before and after morphine injection across the two time points. *p < 0.05.

To assess changes in network coordination, we analysed the temporal synchrony of neuronal activity in S1. On Day 1, acute morphine administration led to a reduction in neuronal synchrony. By Day 7, this desynchronization was even more pronounced (Figure 5), suggesting that morphine tolerance not only affects individual neuronal activity but also disrupts global network coherence. These findings indicate that impaired synchrony in S1 may contribute to altered sensory processing during chronic opioid exposure.

## 4 Discussion

This study reveals that the primary somatosensory cortex (S1) undergoes marked functional reorganization during the development of morphine tolerance. Using a combination of behavioural assays, c‐Fos immunohistochemistry and longitudinal in vivo two‐photon calcium imaging, we show that repeated morphine administration enhances neuronal excitability, reshapes single‐neuron response profiles and disrupts coordinated activity across S1 networks. These findings identify the cortex—not traditionally viewed as central to opioid tolerance—as a dynamic and plastic region involved in the adaptation to chronic opioid exposure.

Our data demonstrate increased c‐Fos expression in S1 following repeated morphine administration, suggesting heightened neuronal activation during tolerance. This aligns with previous reports that morphine modulates activity in cortical areas, including S1, which is critically involved in nociceptive integration. The presence of *μ* ‐opioid receptors (MORs) in S1 neurons of excitatory and parvalbumin positive [7]. Further supports the idea that this region can be directly modulated by opioids. Functional and structural plasticity in S1 has been implicated in chronic pain states [8, 9, 10], but its role in opioid tolerance remains underexplored. Our findings suggest that S1 is not a passive sensory relay during chronic opioid use but an active participant in the development of analgesic tolerance. However, we acknowledge that c‐Fos staining was not combined with a neuronal marker such as NeuN in the present study, which limits the ability to definitively attribute all c‐Fos signals to neurons. Future studies incorporating double immunostaining or cell‐type‐specific labelling will be important to further validate the cellular specificity of these findings.

Using in vivo two‐photon calcium imaging, we found that average neuronal activity—encompassing both excitatory and inhibitory neurons in S1—was not significantly altered within 40 min of acute morphine administration on Day 1. This suggests that the immediate cortical response to morphine may be limited or heterogeneous. Higher temporal‐resolution imaging or separate analyses of excitatory and inhibitory neuronal populations may be necessary to detect more subtle or rapid effects of acute morphine. In contrast, by Day 7, following repeated exposure, we observed a robust increase in both the frequency and spatial extent of calcium transients. These findings indicate that morphine tolerance is associated with heightened cortical excitability.

This result is consistent with prior findings from other regions of the central nervous system. For instance, neurons in the ventrolateral periaqueductal grey (vlPAG) exhibit reduced morphine‐induced inhibition after tolerance develops, resulting in elevated excitability [11]. Similarly, calcium imaging of dorsal root ganglion (DRG) neurons and electrophysiological recordings from the spinal cord show that tolerance is accompanied by diminished inhibitory signalling and sustained excitatory drive [12]. Collectively, these findings support the hypothesis that opioid tolerance reflects a shift in the balance of cortical activity, favouring excitation over inhibition.

Our classification analysis revealed that individual S1 neurons respond to morphine in three distinct ways: excitatory, inhibitory or stable. Notably, these classifications were not static. Over the course of tolerance development, the proportion of excitatory (low‐to‐high) neurons increased, while inhibitory (high‐to‐low) neurons decreased. This shift suggests that the functional identity of neurons may change during chronic opioid exposure. These findings echo earlier electrophysiological studies showing that morphine can both excite and inhibit different subsets of neurons [13, 14]. The observed reclassification of neurons implies dynamic network reconfiguration—potentially reflecting synaptic remodelling, receptor plasticity or homeostatic compensatory mechanisms. The expansion of excitatory neurons may be driven by morphine‐induced downregulation of GABAergic inhibition or enhanced glutamatergic transmission, both of which have been reported in tolerance models.

Beyond changes in single‐neuron activity, our data reveal a reduction in neuronal synchrony within the primary somatosensory cortex (S1) following morphine administration. This desynchronization became more pronounced by Day 7, suggesting a progressive disruption of coordinated network dynamics during the development of morphine tolerance. Such impaired synchrony may degrade the temporal precision of sensory processing and contribute to the altered pain perception observed in chronic opioid exposure. Interestingly, previous work has reported increased synchronous oscillatory discharges in the locus coeruleus following morphine administration in rats [15]. However, to our knowledge, similar effects have not been described in other brain regions, including cortical areas. While neuronal desynchronization is well‐documented in conditions such as epilepsy and neurodegeneration, its role in opioid tolerance remains poorly understood and merits further investigation as a potential network‐level mechanism of sensory dysregulation.

While our study highlights S1 as a site of morphine‐induced plasticity, several limitations remain. First, we did not determine whether these cortical changes causally contribute to tolerance or are a downstream consequence. Optogenetic or chemogenetic manipulation of S1 neurons will be essential to test causal relationships. Second, the downstream circuits mediating the influence of S1 on pain perception or tolerance remain unknown. Future work should explore how S1 outputs interface with thalamocortical or descending modulatory pathways. At the molecular level, identifying the calcium‐dependent transcriptional or epigenetic programmes activated in hyperexcitable S1 neurons could yield targets for therapeutic intervention. Lastly, while this study was conducted in mice, translational work using human cortical imaging is needed to establish clinical relevance.

Our findings identify the somatosensory cortex as a dynamic and plastic component of the neural circuitry underlying morphine tolerance. Chronic morphine exposure induces increased excitability, reclassification of neuronal response types and reduced synchrony within the S1 network. These alterations may contribute to impaired sensory processing and represent a novel cortical signature of opioid tolerance. Understanding how cortical networks adapt to prolonged opioid exposure could reveal new strategies for mitigating tolerance and improving the efficacy of pain therapies.

## Author Contributions

G.Z., W.M. and L.J.X. prepared the manuscript with help from other authors. H.S., Y.U.L. and X.R. conceived and designed this research. G.Z., W.M., L.J.X., B.M. and Z.X. performed the experiments. H.S., Y.U.L. and X.R. contributed to the discussion and data interpretation. H.S., Y.U.L. and X.R. initiated and supervised the project. H.S., Y.U.L. and X.R. are the guarantors of this work and, as such, had full access to all the data in the study and take responsibility for the integrity of the data and the accuracy of the data analysis.

## Funding

This work was supported by the Joint Fund Project of the National Natural Science Foundation of China (Grant No. U21A6002 to H.S.), the National Key R&D Program of China (Grant No. 2023YFA1800100 to Y.U.L.), the National Natural Science Foundation of China (Grant No. 82071188 to Y.U.L., Grant No. 82171209 to X.R.) and the Science and Technology Projects in Guangzhou (Grant No. 2024A03J1256 to Y.U.L.).

## Conflicts of Interest

The authors declare no conflicts of interest.

## Data Availability

The datasets used and/or analysed during the current study are available from the corresponding author on reasonable request.
